# The Impact of COVID-19 on Opioid-Related Overdose Deaths in Texas

**DOI:** 10.3390/ijerph192113796

**Published:** 2022-10-24

**Authors:** Karima Lalani, Christine Bakos-Block, Marylou Cardenas-Turanzas, Sarah Cohen, Bhanumathi Gopal, Tiffany Champagne-Langabeer

**Affiliations:** Center for Health Systems Analytics, UTHealth Houston School of Biomedical Informatics, Houston, TX 77030, USA

**Keywords:** COVID-19, substance use disorder, research, public health

## Abstract

Prior to the COVID-19 pandemic, the United States was facing an epidemic of opioid overdose deaths, clouding accurate inferences about the impact of the pandemic at the population level. We sought to determine the existence of increases in the trends of opioid-related overdose (ORO) deaths in the Greater Houston metropolitan area from January 2015 through December 2021, and to describe the social vulnerability present in the geographic location of these deaths. We merged records from the county medical examiner’s office with social vulnerability indexes (SVIs) for the region and present geospatial locations of the aggregated ORO deaths. Time series analyses were conducted to determine trends in the deaths, with a specific focus on the years 2019 to 2021. A total of 2660 deaths were included in the study and the mean (standard deviation, SD) age at death was 41.04 (13.60) years. Heroin and fentanyl were the most frequent opioids detected, present in 1153 (43.35%) and 1023 (38.46%) ORO deaths. We found that ORO deaths increased during the years 2019 to 2021 (*p*-value ≤ 0.001) when compared with 2015. Compared to the year 2019, ORO deaths increased for the years 2020 and 2021 (*p*-value ≤ 0.001). The geographic locations of ORO deaths were not associated with differences in the SVI. The COVID-19 pandemic had an impact on increasing ORO deaths in the metropolitan Houston area; however, identifying the determinants to guide targeted interventions in the areas of greatest need may require other factors, in addition to community-level social vulnerability parameters.

## 1. Introduction

Drug overdose deaths have been sharply on the rise in the United States since 2013, with age-adjusted rates of synthetic, non-methadone opioid overdose deaths increasing by 1040% from 2013 to 2019 [1]. In the 12 months preceding May 2020, the United States saw the largest one-year increase in overdose deaths ever documented, with the greatest increase from March 2020 to May 2020 [2]. During the following year, the Centers for Disease Control’s (CDC) National Center for Health Statistics reported approximately 100,306 drug overdose deaths in the United States in the 12-month period ending in April 2021. This was an approximate 28.5% increase from the year before [3]. An estimated 75,653 of these drug overdose deaths were a result of opioids, an increase of 56,064 from the year before. Overdose deaths from synthetic opioids (primarily fentanyl), psychostimulants, and cocaine also increased [3]. 

Increases in drug overdoses and drug overdose deaths have been studied at the local and state levels for multiple regions throughout the United States. In a retrospective study using four statewide databases in Rhode Island, Macmadu et al. found a 28.1% increase in drug overdose deaths, with synthetic opioids as a leading cause [4]. Similar findings were observed in Ohio, where sharp spikes in fentanyl overdoses as opposed to heroin were responsible for the majority of overdose deaths [5]. Larger cities, such as San Francisco and Chicago, have added to the findings, noting that in the months after the shelter-in-place order (17 March 2020), they found an increase in drug overdose deaths. San Francisco observed a statistically significant increase in the rate of drug overdoses from 365 deaths in the 8.5 months before to 537 deaths in the 8.5 months after the order (*p* < 0.001) [6]. Like others, Chicago’s Cook County of 5.1 million found that deaths due to fentanyl overdoses increased, observing a significant spike following the stay-at-home order [7]. 

Despite nationwide efforts to combat the opioid epidemic, overdose mortality rates continue to climb. The research on trends in opioid overdoses has identified geographic trends, social trends, economic trends, treatment capacity, and prescribing patterns [8,9,10]. A recent review of the research surmised specific social and demographic characteristics, such as race/ethnicity, gender, age, family composition, and poverty, as important factors related to overdose mortality, indicating socio-demographic determinants as a factor in opioid-related mortality [11]. 

These factors may be measured using an existing tool, the Social Vulnerability Index (SVI), which was developed by the United States Centers for Disease Control and Prevention to identify vulnerable communities [12]. The SVI utilizes census data to determine the relative social vulnerability of every census tract in the United States. Social vulnerability refers to the likely negative effects of external stressors on human health, such as poverty, education level, and geographical location. Environmental and man-made disasters can exacerbate social vulnerability and thus negatively impact a community’s ability to recover from such catastrophic events. The SVI was specifically developed to help determine which communities may need additional assistance and resources before, during, and following natural and/or man-made disasters. The SVI comprises 14 social factors that are grouped into four main thematic categories. Each category receives an individual score of vulnerability, as well as an overall or composite score. The scoring system ranges from zero to 1; scores closer to 1 indicate a higher vulnerability. The SVI categories are as follows: (1) socioeconomic status (poverty, unemployment, income, and education); (2) household composition (aged 65 or older, aged 17 or younger, older than age 5 with a disability, and single-parent households); (3) minority status and language (minority status and speaking English “less than well”); (4) housing and transportation (multi-family structure, mobile home, crowding, no vehicle, and group quarters). 

The bulk of the research on the SVI in the U.S. has focused on natural disasters, such as hurricanes, wildfires, disaster management, and other public health “epidemics,” such as obesity and underage pregnancy [13,14,15,16,17,18]. Internationally, emerging research is applying the SVI to natural and manmade disasters (flash flooding, climate change, etc. [19,20,21,22]) and other health outcomes [23,24,25,26]. The opioid epidemic can be considered a “man-made” disaster, as opioid-related deaths continue to increase, despite efforts to curtail this trend. Although the opioid crisis affects communities in all socioeconomic brackets, research shows disparities in the access to treatment and resources [24]. Recent research using the SVI has shown that communities with a high SVI are more vulnerable to severe effects of COVID-19 [27,28,29]. Emerging research applying the SVI to the opioid epidemic has shown disparities in community-level access to medication for opioid use disorder [24,26]; however, no research—to our knowledge—has applied the SVI to opioid-related overdose mortality. 

Although increases in drug overdose fatalities and, more specifically, opioid overdose fatalities have been reported and studied across the United States since the beginning of the COVID-19 pandemic, little has been studied across the Southern states especially in the largest metropolitan areas, which account for the greatest number of opioid overdose deaths [3]. A report from the Houston Health Department—which represents the fourth largest city in the United States—noted in 2020 that first responders saw a 17% increase in overdoses compared to the same period the year prior. In general, this state’s opioid crisis had been on the rise, with increasing rates of opioid-related overdose deaths despite measures to curb opioid prescriptions [9]. A detailed trend since the pandemic remains unknown for this city, which offers a unique landscape in public health research. Harris County is the third largest county in the United States, and Houston is the fourth most populated and the most racially and ethnically diverse city in the United States. Although Houston is a major metropolitan area, nearly a third of Harris County is unincorporated and 1.21% is classified as rural [30,31]. Within Houston, there are pockets of impoverished neighborhoods in otherwise high SES areas, and because Houston does not have residential zoning laws, its neighborhoods comprise a mix of housing types. Finally, the gentrification of Houston’s historic Black neighborhoods are resulting in more income disparity in otherwise low SES communities [32]. We sought to examine changes in opioid-related overdose deaths in the Greater Houston metropolitan area, reviewing the trends before and after the initiation of the pandemic, while taking a closer look at the geographic visualizations and geospatial distribution of deaths. 

## 2. Materials and Methods

### 2.1. Sources of Data

We utilized four sources of publicly available data for our analysis. The first source was from the Institute of Forensic Sciences (IFS Data). These files included information on deaths occurring from years 2015 to 2021 in the Houston metropolitan area. Variables included dates of birth and death. The second source of data utilized was the Housing and Urban Development (HUD)—United States Postal Service Zip Code Crosswalks developed by HUD’s Office of Policy Development and Research (HUD Data). These are crosswalks of postal zip codes to census tract areas in Harris County, Texas [33]. We then used the Centers for Disease Control and Prevention/Agency data for the Toxic Substances and Disease Registry’s Social Vulnerability Index (SVI), which is maintained by the Geospatial Research, Analysis, and Services Program (GRASP) [12]. The last dataset we utilized was the cartographic boundary file for the State of Texas for from the United States Census Bureau (U.S. Census) from 2010 [34]. This file utilized geographic identifiers (GEOIDs) for area tracking. This study was exempt from the university’s Institutional Review Board. No human subjects were used or considered for this study, and no protected health information was used. All data were retrospective and gathered from publicly available sources. 

### 2.2. Outcome Variable

Our study’s primary outcome variable was the number of opioid-related overdose deaths, defined as a death caused by an overdose where an opioid compound was found as the unique cause of the overdose, or among the list of drugs reported by the toxicology analysis. Our secondary outcome was to describe the location of these deaths by census tract and geographic location as labeled by ordered categories of increased SVIs.

### 2.3. Variables of Study

We merged all IFS data into one file using a unique identifier for each death. Variables of the study included the date of birth and date of death for each decedent, and the city, street, and zip code for where the death occurred. Demographic data included age, gender, and race/ethnicity. Race/ethnicity was classified as White, Black, Hispanic, Asian, or American Indian. The manner of death was categorized as accident, accident/drowning, accident/motor vehicle, suicide, homicide, natural, and undetermined. The place of death was classified as hospital or non-hospital death, and hospitals included health care facilities that were private or public. Non-hospital deaths included deaths that occurred in private residences, motels, hotels, parks, or where the decedent was found in a street, lake, or bayou. We extracted types of opioid or non-opioid drugs reported as the primary or secondary cause of death. 

We utilized the crosswalk files associated with the HUD data to convert the zip codes from the IFS data into coded specific census tract areas. Each census tract area was labeled with its corresponding social vulnerability index category. We utilized the aggregated SVI corresponding to the year 2018, which combines themes of social vulnerability (socioeconomic, household composition, minority status/language, and household type/transportation) as provided by the GRASP. Lastly, we merged the aggregate SVI data for the state of Texas (version 2018) with our working file of IFS data and HUD data by using the census tract variable.

### 2.4. Graphic Representation

After completing our data merging of the IFS data (zip code based) with SVI data (census tract-based) using HUD data (conversion of zip code to census tract), we created the graphics in this paper. We matched the census tract code with the GEOID codes from the cartographic boundary file, based on U.S. Census Bureau instructions, using Tableau© version 2022.2 (https://www.census.gov/programs-surveys/geography/guidance/geo-identifiers.html, accessed on 1 January 2020, Tableau Software, LLC, Seattle, WA, USA). As hospital deaths did not reflect the place of residence of the deceased, these were excluded from the visualizations. We created three maps showing the distribution of opioid-related overdose deaths in the pre–post-pandemic years of 2019, 2020, and 2021. These are dual-axis maps, where the deaths are plotted on a census tract map highlighting areas of socially vulnerability. The size and color of the dots denotes the increase in the number of cases. 

### 2.5. Statistical Analyses

We reported the frequencies and proportions for categorical variables. Frequencies and measures of central tendency were reported for age at death. We constructed a time series panel where data on deaths from IFS data were collapsed by adding up all deaths occurring in the same month and ordering them by month and year. We conducted linear regression analysis to determine the trends between the years of study. For this analysis, we used the observed deaths as the dependent variable and months and years of study as independent variables, using the year 2015 as a reference. Since years 2019 to 2021 were significantly different to our reference year, 2015, we performed an additional analysis for years 2019 to 2021 and used the year 2019 as a reference with the aim of understanding additional differences.

Our statistical analysis showed that the aggregate SVI (version 2018) was not normally distributed; hence, we coded it in quartiles when associated with the corresponding geographic areas of the cartographic boundary files from the U.S. Census. Demographic characteristics associated with differences in SVI quartiles were evaluated with Pearson’s Χ square when categorical and ANOVA when continuous. A probability value of <0.05 (two-tailed) was considered statistically significant for all tests. We performed all statistical analyses using Stata IC 15 (StataCorp LLC, College Station, TX, USA). Tableau© version 2022.2 (Tableau Software, LLC) was utilized for the geospatial analyses and geographic visualizations.

## 3. Results

There were 2660 documented opioid-related overdose deaths in the Houston metropolitan area between 1 January 2015 and 31 December 2022. Of those, 61 (2.29%) had an unknown age and 15 (0.56%) were under the age of 18. The mean (standard deviation, SD) age at death was 41.04 (13.60) years. The majority of the deaths were male (68.12%) and White (62.77%). The proportion of deaths increased by 182.72% from 2015 to 2021, with a notable spike in the years 2020 and 2021. The majority of the deaths were declared accidental (94.4%), followed by suicide (4.47%). Over 75% of the deaths occurred out of the hospital, as shown in Table 1.

Non-opioid substances were present in most of the deaths, with 68.34% (1818) of cases overdosing with more than two substances, 24.89% (662) of cases reported two substances, and only 10.68% (284) of cases had only one substance (opioid) reported. Of the other substances detected, stimulants (55.53% of cases) and benzodiazepines (54.43% of cases) were the most common, followed by antihistamines (11.99%) and sedatives (6.54%). Heroin was the opioid present in most cases (43.35%), followed closely by fentanyl (38.46%), and distantly by hydrocodone (19.92%) and oxycodone (8.61%), as shown in Table 2. 

**Figure 1:**
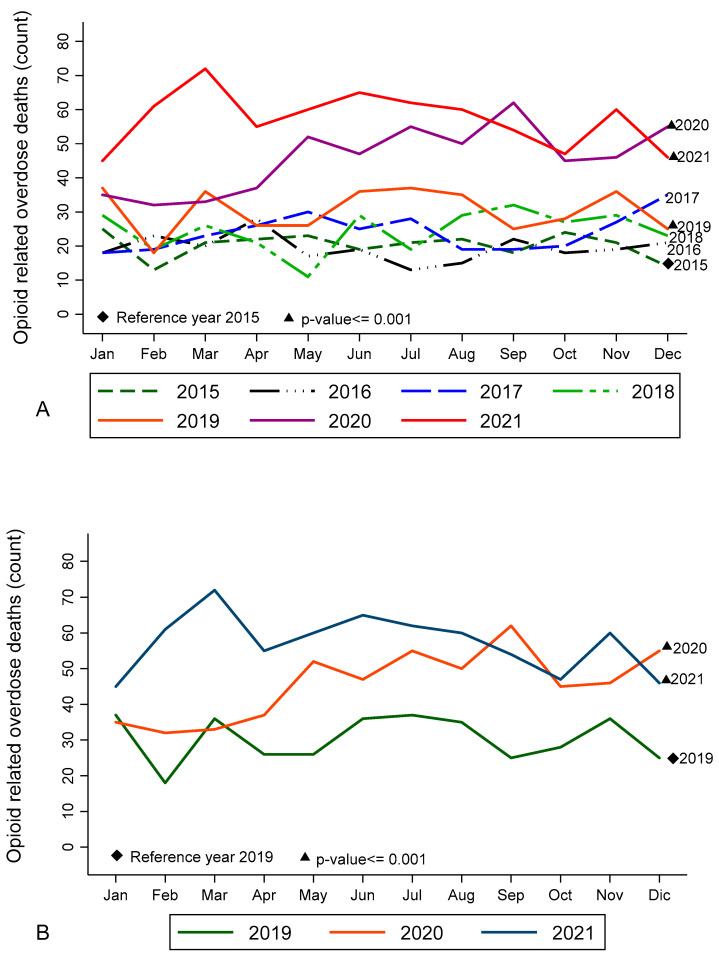
Time trends of opioid-related overdose deaths in the metropolitan Houston area from 1 January 2015 to 31 December 2021. Source: Harris County Institute of Forensic Sciences. Compared to 2015, the trends showed a significant increase in deaths after 2018 (*p*-value < 0.001), which is shown in solid lines. (See subfigure **A**). Compared to year 2019, the trends showed a significant increase in deaths for 2020 and 2021 (*p*-value < 0.001). (See subfigure **B**).

An analysis of the opioid overdose trends showed significant increases during 2019 to 2021 when compared to 2015 (Figure 1A). Significant increases were also seen between 2019 (a pre-pandemic year), 2020, and 2021 (Figure 1B). No trend differences were observed between the years 2020 and 2021.

While the overall opioid-related overdose mortality increased across the SVI quartiles, there were slightly more deaths observed in areas where the SVI was high during the COVID-19 pandemic, although the increment was not linear along the quartiles. We found the mean age at death increased across each SVI quartile: where the SVI was low, the mean age was 39.94 years; where the SVI was high, the mean age was 42.61 years. 

**Figure 2:**
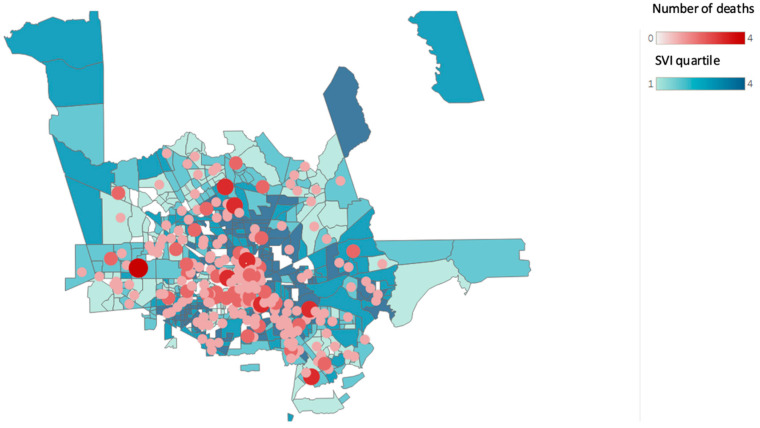
Non-hospital opioid-related overdose deaths and Social Vulnerability Index (SVI) according to census tract for the year 2019. (Source: Harris County Institute of Forensic Sciences, 2015–2021, U.S. Census Bureau; Centers for Disease Control and Prevention and the Agency for Toxic Substances and Disease Registry; and the U.S. Department of Housing and Urban Development Zip Code Crosswalk). The figure shows the total opioid-related overdose deaths based on the residential zip code counts to 270 for the year 2019.

**Figure 3:**
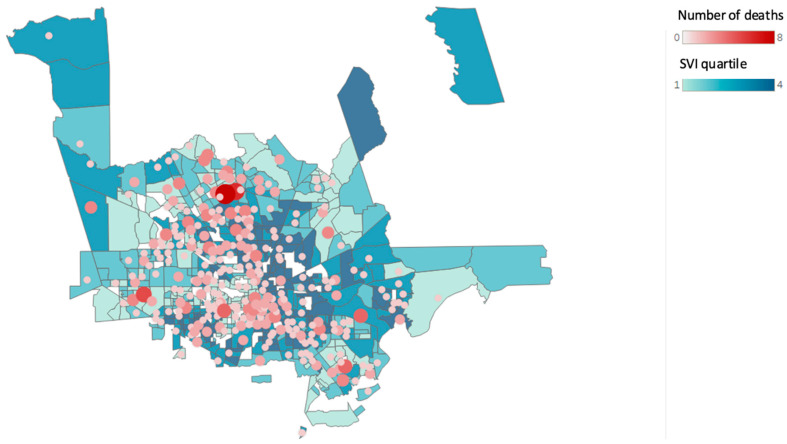
Non-hospital opioid-related overdose deaths by Social Vulnerability Index (SVI) according to census tract for the year 2020. (Source: Harris County Institute of Forensic Sciences, 2015–2021, U.S. Census Bureau; Centers for Disease Control and Prevention and the Agency for Toxic Substances and Disease Registry; and the U.S. Department of Housing and Urban Development Zip Code Crosswalk Files). The figure shows the total opioid-related overdose deaths based on the residential zip code counts to 450 for the year 2020.

**Figure 4:**
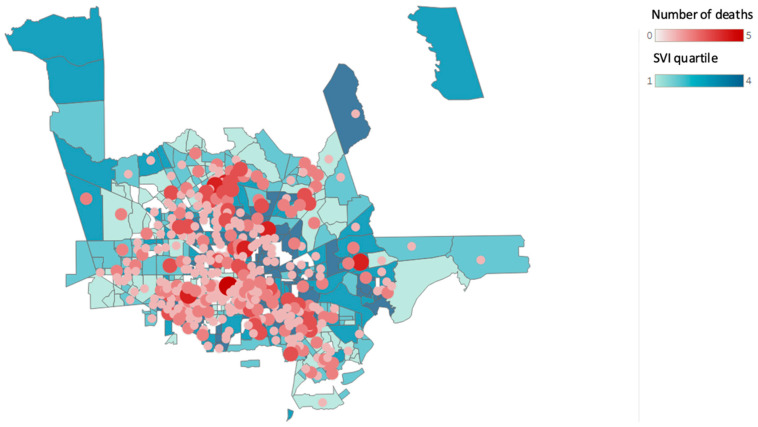
Non-hospital opioid-related overdose deaths by Social Vulnerability Index (SVI) according to census tract for the year 2021. (Source: Harris County Institute of Forensic Sciences, 2015–2021, U.S. Census Bureau; Centers for Disease Control and Prevention and the Agency for Toxic Substances and Disease Registry; and the U.S. Department of Housing and Urban Development Zip Code Crosswalk Files). The figure shows the total opioid-related overdose deaths based on the residential zip code counts to 567 for the year 2021.

Figure 2, Figure 3 and Figure 4 show the changes in geographical location of the non-hospital opioid-related overdoses from 2019 to 2021.

## 4. Discussion

Our study revealed trends in opioid-related overdose deaths in the Houston metropolitan area, before and during the initial period of the pandemic. January 2019 and January 2020 saw a similar number of opioid-related overdose deaths, with a slight reduction in deaths noted in February 2019. The declaration of a local disaster was issued by government officials on 11 March 2020 [35]. Shortly thereafter, food establishments and bars were ordered to be closed, and a stay-at-home order was in effect by 24 March 2020, closing schools and all non-essential businesses. Though one study found buprenorphine prescribers and prescriptions remained stable during this time, in-person ambulatory care decreased significantly as a result of the stay-at-home order [36]. In April 2020, the pandemic declarations extended the stay-at-home order, closed public parks, released low-risk offenders from county jails, and limited outdoor gatherings. There were significant increases in opioid-related deaths, specifically after these social-distancing restrictions were enacted.

Our study shows that opioid-related overdose deaths steadily increased during the summer of 2020. Throughout June, July, and August of 2020, the pandemic restrictions were partially lifted and then reinstated, with non-essential businesses closing again in late June due to high positivity rates. In September 2020, there was a dramatic increase in mortality of 3.87%. Houston area hospitals and emergency departments reached full occupancy and hospitals began to reassess capacity by diverting patients [37]. Our research is consistent with other research on the impacts of COVID-19 on opioid overdose deaths. Significant increases in opioid-related mortality were seen across the United States [38,39], with increases occurring during social distancing and continuing during reopening [40]. Although substance use treatment was considered an essential service during the height of the pandemic, staff and counselors who had a positive COVID-19 test or were exposed to a confirmed case were required to stay home for up to two-weeks, leaving many treatment centers understaffed and individuals already at risk of an overdose without a back-up plan, potentially affecting mortality [40]. Individuals released from jail are over 12 times more likely to experience a fatal overdose than the general population [41]. The data from Harris County shows that the jail population decreased by approximately 1500 inmates between March and April 2020, and our data show overdose deaths increasing sharply during this period. A similar trend is noted in the first quarter of 2021: as the Harris County Jail population decreases, overdose deaths increase [42]. The Harris County jail followed the trends seen in jails across the United States, with the incarcerated population sharply declining in March 2020, and overdose rates rising.

Our data show that overdose deaths reached a record all-time high during March 2021 in the metropolitan Houston/Harris County region. During the same time, Texas faced record-low temperatures and the state’s power grid failed under the exceptional strain, leaving millions of Texans without electricity, heat, or water for multiple days [43]. Opioid use can increase the risk of cardiac events, and preliminary studies have found a correlation between cold temperatures and an increased risk of opioid overdose [44,45]. Like hospitals across Houston, Harris County’s medical examiner services were overwhelmed during the height of the pandemic, and the winter storm added further complications. Compared to the week before, the week of the power-outage saw an 18% increase in deaths across Texas that were not related to COVID-19 [46]. County-specific variations in data reporting for vital statistics offices created challenges in extricating the exact cause of this drastic rise in deaths. The winter storm of 2021 created a crisis within a crisis, and without complete forensic investigations, the full scope of opioid-related mortality during this time cannot be determined. 

Although previous research shows that certain social characteristics are associated with an increased risk of opioid-related fatalities, our data show overdose deaths occurring in the middle to upper-income areas [47]. Due to Houston’s extraordinary demographic and economic diversity, many neighborhoods are scattered with concentrations of poverty. Since zip codes comprise multiple census tracts, it is possible that zip code-level data are not sensitive enough to account for income disparities. Harris County has a median household income of USD 63,022 and an average income of USD 33,459 [48]. While we expected to see a direct linear relationship between a high SVI and overdoses, the top 20 zip codes that had the highest rates of opioid-related overdose deaths were not in low socioeconomic zip codes. This study does not support the bulk of research on community-level opioid overdose risk factors that show a low socioeconomic status, education, poverty, and unemployment as directly relating to overdose risk. While individual vulnerability factors may be related to overdose rates, the reliability of an aggregate SVI to predict community-level overdose risk is not supported, even during a natural disaster. These results support the need for additional research on the micro-level factors that indicate pockets of vulnerability within larger communities. Undeterred by national and local efforts to manage the opioid crisis, a fourth wave driven by fentanyl and stimulants is predicted [49]. Our study shows there are other complex relationships that communities need to consider, including polysubstance use and access to treatment [50]. 

Our data found a positive linear relationship with the aggregate SVI and age of death. The average age of death in low-SVI areas was 39.59 years, which increased steadily across SVIs to 43.21 years in high-SVI areas. Similarly, race/ethnicity was found to be correlated with SVI, with more non-White individuals in areas with a higher SVI. As race/ethnicity is a factor in calculating the SVI, this observation was not unexpected. Opioid-related overdose has increased in all age groups and in all racial/ethnic groups, largely driven by the increased availability of synthetic opioids [51]. However, the research has shown differences regarding initiation, polysubstance use, opioid type, and the mode of use among different races/ethnicities, indicating that there is no uniform solution [52]. Harris County and Houston are among the most racially and ethnically diverse metropolitan areas in the U.S., with varying levels of segregation throughout the area [53]. Our research underscores the importance of local and community-based interventions for opioid use disorder, particularly in large metropolitan areas that are racially/ethnically, geographically, and economically diverse. The community-level factors that contribute to overdose vary; therefore, interventions should be tailored to community-level solutions. 

### Strengths and Limitations

There have been several studies on the impact of COVID-19 on opioid overdose deaths across the United States; however, this is the first study in the state of Texas to utilize multiple databases and evaluate sociodemographic variables during the pandemic. Additionally, we were able to extend the period of review to cover the entire years of 2020 and 2021, thereby showing not only the immediate impact of the pandemic, but the prolonged impact on opioid overdose deaths. Finally, to our knowledge, this is the first study of its kind to correlate the place of opioid overdose death with the SVI. The data used for this analysis were specifically reported by the Institute of Forensic Sciences and may have been under-reported or misclassified. Our study does not address non-fatal overdoes and naloxone administration by EMS, and does not capture naloxone purchases at pharmacies, and, therefore, may fall short of recognizing the full scope of opioid-related overdoses in the Houston Metropolitan area. Due to the study’s design and the nature of the decedents, we had data on the location of death—some of which occurred within the hospital. For an accurate SVI analysis, we had to exclude roughly 20% of the data as there was no place of residence or where the initial overdose occurred. Finally, retrospective data are subject to confounding; thus, these results can only show associations. Extricating the exact causes of the drastic rise in opioid-overdose deaths in the Houston metropolitan area is difficult; however, it is possible that the impact of the COVID-19 pandemic and related stressors on existing economic and social conditions directly impacted opioid-related overdose rates in Houston and across the United States.

## 5. Conclusions

Opioid overdose deaths are increasing across the United States, including in the Greater Houston metropolitan area. Our data showed increases in pre-pandemic opioid overdose deaths that were further exacerbated during the pandemic. Developing tools to identify communities of greatest need is essential to save lives through preventive and harm-reductive measures. Research has previously shown that social-demographic factors have been associated with opioid overdose deaths; however, we have found that using community level data may not be sufficient for identifying the neighborhoods of greatest need. For our analysis, we used aggregate SVI data and there may be benefits in looking at the index on a detailed level, either by category or individual variable. Furthermore, the SVI may need to be combined with other community parameters, possibly including access to opioids, both prescribed and illicit, and the frequency of interruption in the city-provided services. More work is needed to prospectively identify geographical hotspots. Future work may include compiling additional data sources and the use of machine learning to identify key variables and develop a predictive model. 

## Figures and Tables

**Table 1:** Demographic characteristics of 2660 patients who died of opioid-related overdoses in metropolitan Houston area between 1 January 2015 to 31 December 2021.

Characteristics	N ^1^
Sample Size	2660 (100%)
Gender	
Male	1812 (68.12%)
Female	848 (31.88%)
Race/Ethnicity	
White	1671 (62.82%)
Black	467 (17.56%)
Hispanic	464 (17.44%)
Asian	56 (2.11%)
American Indian	2 (0.08%)
Year	
2015	243 (9.14%)
2016	233 (8.76%)
2017	289 (10.86%)
2018	294 (11.05%)
2019	365 (13.72%)
2020	549 (20.64%)
2021	687 (25.83%
City	
Houston	1849 (69.51%)
Pasadena	122 (4.59%)
Spring	106 (3.98%)
Other	586 (21.92%)
Manner of death	
Accident	2512 (94.44%)
Suicide	119 (4.47%)
Accident/drowning	11 (0.41%)
Homicide	4 (0.15%)
Natural	3 (0.11%)
Accident/MVA ^2^	1 (0.04%)
Undetermined	10 (0.38%)
Place of death	
Not in-hospital	2096 (78.80%)
Hospital	564 (21.20%)

^1^ Note: Percentages may not add up to 100 due to rounding. ^2^ MVA, Motor vehicle accident. Source: Harris County Institute of Forensic Sciences.

**Table 2:** Types of substances reported in 2660 opioid-related overdose deaths in the metropolitan Houston area from 2015–2021.

Type of Substances Reported	N ^1^
Total deaths in the period of study	2660 (100%)
Opioids	
Heroin	1153 (43.35%)
Fentanyl	1023 (38.46%)
Hydrocodone	530 (19.92%)
Oxycodone	229 (8.61%)
Tramadol	169 (6.35%)
Other substances	
Alprazolam	864 (31.26%)
Cocaine	735 (27.63%)
Methamphetamine	739 (27.78%)
Ethanol	532 (20.0%)
Diazepam	214 (8.05%)

^1^ Rows are independent of each other. Frequencies and percentages may sum more than total deaths and 100% because cases may have had more than one type of substance reported. Source: Harris County Institute of Forensic Sciences.

## Data Availability

Data from this study may be obtained by reasonable request by contacting the corresponding author.
